# Non-Surgical Treatment of Periodontal Disease in a Pregnant Caucasian Women Population: Adverse Pregnancy Outcomes of a Randomized Clinical Trial

**DOI:** 10.3390/ijerph16193638

**Published:** 2019-09-27

**Authors:** Leticia Caneiro-Queija, Jose López-Carral, Pablo Martin-Lancharro, Jacobo Limeres-Posse, Pedro Diz-Dios, Juan Blanco-Carrion

**Affiliations:** 1 Medical-Surgical Dentistry Research Group (OMEQUI), Health Research Institute of Santiago de Compostela (IDIS), University of Santiago de Compostela (USC), 15782 Santiago de Compostela, Spain; leticiacaneiro@gmail.com (L.C.-Q.); carralami@gmail.com (J.L.-C.); pablo.martin.lancharro@sergas.es (P.M.-L.); pedro.diz@usc.es (P.D.-D.); jblanco@blancoramos.net (J.B.-C.); 2 Department of Obstetrics and Gynecology, Clinic University Hospital of Santiago de Compostela, 15782 Santiago de Compostela, Spain; 3 Special Needs Unit, School of Medicine and Dentistry, Santiago de Compostela University, 15782 Santiago de Compostela, Spain

**Keywords:** preterm birth, low birth weight, pregnancy, periodontal disease

## Abstract

Aim: To analyze if non-surgical treatment of periodontitis in a pregnant Caucasian women population can reduce adverse pregnancy outcomes. Methods and results: A parallel randomized clinical trial was designed and approved by the Ethical Committee of Sanitary Area Santiago-Lugo, Spain (registration number: 2016/451). Forty patients with periodontitis stage II grade B were randomly allocated to receive either comprehensive non-surgical periodontal therapy (test group; *n* = 20) or professional tooth cleaning (control group; *n* = 20) before 24 gestational weeks. Randomization was computer-generated by the statistic program Epidat v.4.1 and allocation was performed using sealed opaque envelopes. Clinical measurements and peripheral blood samples for biochemical variables were collected at baseline, in the middle of second trimester before non-surgical treatment, and in the third trimester. Microbiological samples were collected in the second and third trimester. A statistically significant reduction was verified in all clinical and microbiological parameters after periodontal treatment in the test group. No significant differences were observed for the rest of the variables, including preterm birth and/or low birth weight. No adverse events related to periodontal treatment were reported. Conclusions: Non-surgical periodontal treatment in Caucasian patients with periodontitis stage II grade B did not significantly reduce the risk of adverse pregnancy outcomes.

## 1. Introduction

Adverse results of pregnancy, such as preterm birth or low birth weight, can be the cause of mortality and increased morbidity in neonates [1]. Worldwide, preterm birth is the second most common factor of death in children under 5 years old [2]. 

The prevalence of preterm births varies depending on the country (ranged from 5% in some European countries to 18% in some African countries). Blencowe et al. [3] identified data from 99 countries and estimated the 2010 global prevalence at 11.1% (95% CI = 9.1–13.4%). The preterm birth rate in the 1980s was not vastly different than the current preterm birth rate [4], and it remains an important public health priority worldwide [5].

Periodontitis is an inflammatory infection disease affecting the tooth-supporting tissues, initiated by a biofilm with gram-negative anaerobic microorganism predominance and mediated by the inflammatory response of the host. It is also liable to induce systemic inflammation and consequently modify the normal development of pregnancy [6]. During pregnancy, due to hormonal changes, there may be a tendency towards periodontal disease, in particular, there is an increase of anaerobic gram-negative bacteria such as *Fusobacterium nucleatum*, *Treponema denticola*, *Tannerella forsythia*, *Campylobacter rectus*, *Eikenella corrodens,* and *Selenomonas sputigena* [7].

Infections account for 75% of premature births and/or low birth weight, while subclinical infections begin to be considered as an important cause of very premature birth (before the 30th week of gestation). However, in more than 50% of cases, the etiological factors of prematurity are unknown [8]. Oral infections might be considered one of these factors, since commensal bacterial species of the oral cavity colonize the fetoplacental unit of women with full-term gestation and with adverse pregnancy outcomes [9]. The bacterial species most strongly associated with adverse pregnancy outcomes include: *Fusobacterium nucleatum*, *Campylobacter rectus*, *Porphyromonas gingivalis,* and *Bergeyella* spp. [10].

Two main routes have been proposed to trigger an inflammatory response in the fetoplacental unit; the direct route, in which the oral microorganisms and/or their components reach the fetoplacental unit by hematogenous dissemination of the oral cavity or by an ascending route through the genitourinary tract; and the indirect route, in which the inflammatory mediators produced in the periodontal tissues circulate and impact the fetoplacental unit. A large number of studies associate an increase in the levels of local and systemic inflammatory markers with adverse pregnancy outcomes [11]. As well as this, hormonal changes during pregnancy, due to elevated levels of estrogen and progesterone, increase vascular permeability in gingival tissues and, as a consequence, bacteria and/or their products can diffuse more easily [12].

The increase in maternal serum levels of proinflammatory cytokines, such as IL-1, IL-6, IL-8, and TNF-α have been associated with prematurity and low birth weight [13,14]. C-reactive protein, which is an acute-phase reactant synthesized by the liver in response to proinflammatory cytokines, has also been associated with prematurity [15].

Cohort studies have shown a relationship between periodontal disease, premature delivery, and low birth weight [16,17,18], as it has been confirmed in some systematic reviews [19,20,21,22]. On the other hand, clinical trials have studied the effect of periodontal treatment on adverse pregnancy outcomes and showed controversial results [8,21,22,23,24,25,26,27,28,29,30,31]. This is probably because there is a marked heterogeneity between studies in terms of the population´s characteristics (race, age, etc.) as well as the periodontitis definition applied. Finally, it should be noted the conclusions reached in the Nine European Workshop on Periodontology in 2012 indicating that, up to now, it is not possible to affirm that periodontal treatment reduces the rates of premature delivery or birth of underweight children [32]. However, these studies have provided that non-surgical periodontal treatment, when performed on pregnant women, is safe for the mother and the fetus [32,33]. 

Our hypothesis was that periodontitis has an effect on pregnancy outcomes, such as preterm birth and/or low birth weight. Therefore, the aim of the current study is to analyze if non-surgical treatment of periodontitis, using the definition suggested by the EFP-AAP Consensus Meeting [34], can reduce adverse pregnancy outcomes in a pregnant Caucasian women population.

## 2. Material and Methods

### 2.1. Study Design 

A parallel randomized clinical trial was designed to test whether the non-surgical periodontal treatment provided during pregnancy affected gestational age at delivery and birth weight. Data were gathered on the pregnant women at three visits: at the end of the first trimester (16 weeks of pregnancy), in the middle of second trimester (22–24 weeks of pregnancy), and at the third trimester (33–36 weeks of pregnancy), as usually recommended follow-up visits in pregnant woman. No relevant changes were performed after trial commencement.

### 2.2. Study Population

**Figure 1:**
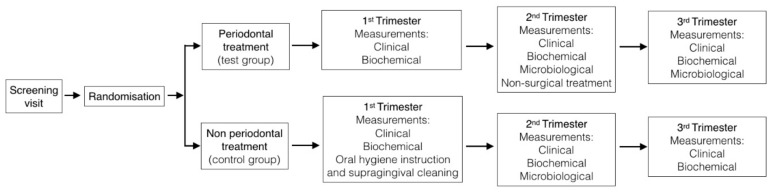
Research outline.

Pregnant women who were seen for prenatal care at the University Hospital of Santiago de Compostela (Spain) were invited to participate in the study when they attended the hospital to undergo the first gynecological clinic appointment between June 2017 and May 2018. During this screening visit, a full-mouth periodontal examination was performed and patients with a periodontitis diagnosis [34] were invited to participate. The sample size was done with PASS v.12 (NCSS, LCC; Kaysville, UT, USA) with an 80% power and a significance level of 0.05, sample size was established as 40 patients. The patients who were included in the study were divided in two groups (test and control) according to a previous computer-generated randomization (generated by the statistic program Epidat v. 4.1) and allocation ratio 1:1 (using sealed opaque envelopes labeled with a study number and containing the group allocation opened by examiner after baseline examination). The test group corresponded to patients with non-surgical periodontal treatment, and the control group consisted of patients without periodontal treatment (Figure 1). All potential participants signed an informed consent form to participate in the study. This protocol was approved by the Ethical Committee of the Sanitary Area Santiago-Lugo, Spain (reference: 2016/451). 

### 2.3. Inclusion/Exclusion Criteria

Inclusion criteria were Caucasian pregnant women (gestational age ≤16 weeks), aged 18 to 40 years, with more than 20 natural teeth and diagnosis of periodontitis. 

Exclusion criteria were multiple gestation; previous preterm low birth weight; more than 1 previous miscarriage or one greater than 18 weeks; diabetes; hypertension; alcoholism; drug abuse; human immunodeficiency virus (HIV) infection; heart disease, kidney disease or liver disease; recurrent cystitis; viral infections; venereal infections; toxoplasmosis; and/or tobacco smoking.

### 2.4. Maternal Characteristics

Demographic data and medical history were assessed by interview during the first visit (16th gestational week). The interview included items on age, educational level (primary or less, high school, and university), area of residence (rural or urban, depending on the number of inhabitants), body mass index (BMI), marital status (single, common-law partner, married), and obstetric history (number of previous pregnancies, previous miscarriage). 

The following variables were recorded shortly after delivery: newborn weight, newborn sex, duration of pregnancy, and type of delivery (vaginal or caesarean). 

### 2.5. Periodontal Measurements

Full-mouth periodontal examinations were performed by one examiner. The weighted κ values for intra-examiner calibration were 0.82 (CI 95% = 0.68–0.98). Periodontal clinical measurements were recorded at enrolment and repeated at 2nd and 3rd trimester. Exams included all teeth present in the mouth (excluding third molars). A plaque score [35], periodontal pocket depth (PPD) (six sites per tooth), clinical attachment level (CAL) and bleeding on probing (BOP) [35] (six sites per tooth) were registered. All scores were measured with a manual periodontal probe UNC-15 (Hu-Friedy, Chicago, IL, USA). Periodontitis was defined according to the case definitions of the World Workshop in Periodontology in 2018 [34]:

1. Interdental CAL was detectable at ≥2 non-adjacent teeth, or
2. Buccal or oral CAL ≥3 mm with pocketing ≥3 mm was detectable at ≥2 teeth but the observed CAL could not be ascribed to non-periodontitis-related causes such as: (1) gingival recession of traumatic origin; (2) dental caries extending in the cervical area of the tooth; (3) presence of CAL on the distal aspect of a second molar and associated with malposition or extraction of a third molar; (4) an endodontic lesion draining through the marginal periodontium; and (5) the occurrence of a vertical root fracture.

### 2.6. Biochemical Variables

A peripheral blood sample was collected from each subject with venipuncture using a vacuum tube (Vacutainer, Nippon Becton Dickinson Tokyo, Japan). Samples were centrifuged at 2500 rpm for 10 minutes and the serum obtained was dissociated in a plastic tube. At the time of the analyses, the serum concentration of interleukin-6 (IL-6), interleukin-8 (IL-8), tumor necrosis factor-α (TNF), and fibrinogen were determined using commercially available enzyme-linked immunoassays (ELISA). 

### 2.7. Microbiological Variables

In the middle of the second trimester (22–24 weeks of pregnancy) before non-surgical periodontal treatment and before the third trimester, four sites (those with the deepest probing depth) were selected, and two consecutive paper points were inserted and kept in place for 10 s. All paper points were pooled in a vial with reduced transport fluid (RTF) and transferred to the laboratory within 12 h [36].

The obtained samples were dispersed (30 s of vortex), serially diluted and processed for culture by inoculation on two different media: blood agar medium (no 2; Oxoid Ltd., Basingstoke, UK), with horse serum at 5% and with hemin (5mg/L) and Dentaid-1 medium [37]. The blood agar plates were studied after 14 days of anaerobic incubation (80% N_2_; 10% H_2_; 10% CO_2_) at 37 °C, and after 3–5 days at 37 °C in air with 5% CO_2_.

*Porphyromonas gingivalis, Prevotella intermedia, Tannerella forsythia, Parvimona micra, Campylobacter restus, Fusobacterium nucleatum, Eikenella corrodens,* and *Capnocytophaga* spp., were identified based mainly on their colony morphology and further confirmed with different specific chemical tests. Counts for every bacterial species were obtained and the percentage relative to the total flora calculated. *Aggregatibacter actinomycetemcomitans* were grown on Dentaid-1 medium plates, and identified based on colony morphology and catalase reaction.

### 2.8. Periodontal Treatment

The test group patients received non-surgical periodontal therapy that was completed by the end of week 24 of gestation. This consisted of oral hygiene instructions, followed by mechanical supra and subgingival scaling and root planning (SRP) with Gracey curettes (Hu-Friedy, Chicago, IL, USA). Periodontal therapy was performed over two 1-hour sessions. Periodontal treatment was performed by one periodontist (L.C.) in the Department of Periodontology at the University of Santiago de Compostela (Spain).

The control group received professional tooth cleaning (oral hygiene instructions and supragingival cleaning of all teeth) at the second-trimester visit. All control participants were offered the opportunity to attend for non-surgical periodontal therapy postpartum.

### 2.9. Outcomes

Primary outcomes of the present study were preterm birth and low birth weight. Following the World Health Organization criteria, preterm birth was defined as a delivery at <37 weeks of gestation (gestational age determined by last menstrual period and ultrasound fetal measurement) and low birth weight was defined as a newborn weight of 2500 gr or less. Secondary outcomes were those related to the efficacy of non-surgical periodontal therapy on clinical, biochemical and microbiological variables.

### 2.10. Statistical Analysis

Descriptive analysis was calculated for each variable (mean values, standard deviation). Kolmogorov–Smirnov test was performed to test the normality of the variables. Relationship between each variable and preterm birth/low birth weight were analyzed. All variables were also compared between women with and without treatment. A t-student test was performed for continuous variables whilst A chi-square test was used to analyze categorical variables. Non-normal distribution variables were analyzed by a Mann–Whitney U test for continuous variables and chi-square test for categorical variables. Multivariate analysis was performed using ANCOVA test, with pregnancy and newborn weight as dependent variables with age, body mass index as a covariable, and treatment as an independent variable. Statistical significance was established at the 95% confidence level and *p*-values <0.05 for all analyses were selected to be statistically significant. SPSS for Windows (SPSS Inc. version 20.0, Chicago, IL, USA) was used for all the statistical analyses. 

## 3. Results

**Figure 2:**
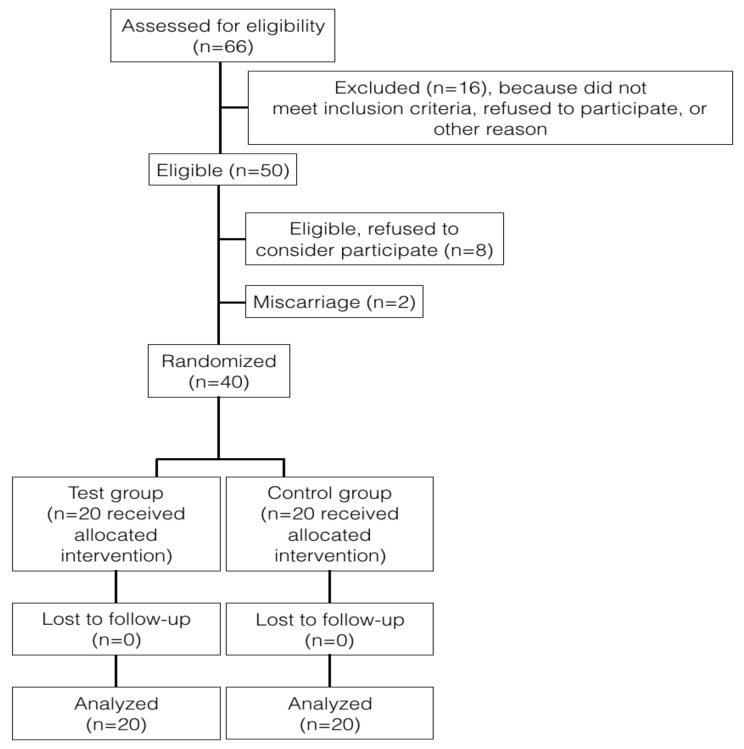
Flow chart to show participants progress through the study.

Initially, 66 women were examined and 50 met the inclusion criteria. Eight rejected to participate and two had a miscarriage. Finally, 40 women with periodontitis stage II grade B complied with all the visits, 20 received non-surgical treatment (test group), and 20 received no treatment was performed (control group), as shown in Figure 2. No adverse events related to periodontal treatment were reported.

Demographic characteristics of the study sample are shown in Table 1. No statistically significant differences were observed between groups. The study population was 32.00 ± 4.27 years old for the test group and 32.25 ± 4.21 for control group; the majority lived in a rural residence, were married, had finished elementary school, and were her first pregnancy and only a 5% had a previous spontaneous abortion. 

Table 2 depicts the periodontal status of the study population. Selected pregnant women presented a mean number of teeth in the test group of 26.95 ± 1.21 and 26.32 ± 2.77 in control group, with no significant differences between them. Bleeding on probing, probing pocket depth, and clinical attachment level were statistically higher for the test group in the 1st trimester. However, after non-surgical periodontal treatment these parameters were reversed, and a significative reduction was verified in the 3rd trimester in this test group.

Table 3 presents the cytokine levels in peripheral blood according to experimental groups and experimental periods. No significant differences were observed for fibrinogen, IL-6, IL-8, and TNF-α between groups.

The microbiological findings from 40 pregnant women, showed high proportions of detection of *Porphyromonas gingivalis, Fusobacterium nucleatum, Prevotella intermedia*, and *Tannerella forsythia*. No statistically significant differences were observed between the control and test group. Table 4 shows proportions of different bacterial species in test group before (2nd trimester) and after treatment (3rd trimester), this difference being statistically significant in some bacteria.

The mean gestational duration was 38.25 ± 2.88 weeks for the control group and 37.72 ± 6.04 weeks for the test group. Mean weight at birth were 3012.59 ± 415.42 g for the control and 3249.48 ± 473.54 g for the test group. No statistically significant differences were observed between groups for these variables.

Table 5 shows neonatal and obstetric outcomes. No significant differences in preterm birth rates were observed between groups (OR = 0.28; CI = 0.02–2.98). Similarly, the occurrence of newborns with low birth weight was not significantly different between groups (OR = 0.28; CI = 0.02–2.98).

Table 6 and Table 7 described pregnancy time (weeks) and newborn weight (kg) as dependent variables and the relation with age, body mass index, and treatment (independent variables). No significant differences were obtained.

## 4. Discussion

The main objective of this study was to evaluate the effects of non-surgical periodontal treatment in pregnant women, carried out during the second trimester of pregnancy, on the rates of preterm birth and low birth weight, and to assess the clinical, biochemical, and microbiological changes. The effect of periodontal intervention on pregnancy outcomes did not show a reduction in the risk of preterm birth and low birth weight in the test group compared to the control group. However, we can assume as clinically relevant the protector role of periodontitis treatment during pregnancy (ANCOVA analysis), due to the significant periodontal health improvement in the test group. Pregnancy is a short period of time in order to evaluate the systemic impact of a long term periodontal microbial infection and related inflammatory responses. 

This finding is supported by previous studies such as Oliveira et al. [31], Offenbacher et al. [30], and Michalowicz et al. [38]; these authors demonstrated that the treatment of periodontitis in pregnant women was safe and effective for periodontal disease, but did not reduce the incidence of premature delivery and low birth weight. The meta-analysis conducted by Baccaglini in 2011 [39] including eleven high quality randomized controlled trials performed on 6558 pregnant women, also showed no evidence to support that non-surgical periodontal treatment during pregnancy prevents premature birth or other adverse pregnancy outcomes. 

Conversely, some papers have suggested a positive effect of non-surgical periodontal treatment both in the periodontal condition of the patient and in the adverse results of pregnancy. Offenbacher et al. [24] showed that periodontal treatment reduced 3.8 times the rate of premature births; this may be due to the fact that many of the included patients had a disadvantaged economic status and had previously had preterm births (>12 weeks), therefore, this study population represented a high-risk group. Jeffcoat et al. [23] showed that successful periodontal therapy reduced the risk of premature delivery in a group of African-American women who had never visited a dentist, but their results cannot be extrapolated to other populations with different characteristics. Radnai et al. [25] also showed that periodontal treatment had a positive pregnancy outcome in a demographically homogenous Caucasian European population, but these women had an initial localized chronic periodontitis. Tarannum et al. [26] provided evidence that non-surgical periodontal therapy can reduce the risk of preterm birth/low birth weight, but the study population was Indian, belonged to low socio-economic strata (based on occupation), had low education levels, and patients with a history of alcohol/tobacco consumption were not excluded. Finally, López et al. [8] also demonstrated the positive effect of periodontal treatment in adverse pregnancy outcomes in patients diagnosed with gingivitis and periodontitis, but the periodontal treatment included a daily chlorhexidine rinse at 0.12% and women with urinary tract infections were treated with antibiotics (an exclusion criteria in most studies).

When the clinical variables were analyzed, we have to consider that bleeding on probing, probing pocket depth, and clinical attachment level were statistically higher for the test than for the control group in the 1st trimester, however both groups were diagnosed with periodontitis stage II, grade B [34]. These clinical parameters remained stable throughout the gestational period in the control group and after treatment in the test group, there was a significative decrease in the percentage of bleeding on probing, plaque index, average probing pocket depth, and clinical attachment loss. These findings for the control group seems to have no correlation with plaque accumulation since it has been shown that the hormonal changes that occur during pregnancy are a modifying factor of bacterial plaque [40]. Santa Cruz et al. [9] showed that periodontal clinical status was not associated with adverse pregnancy outcomes in a Caucasian Spanish population with a medium–high educational level.

Biochemical markers increased in the third trimester following the basic periodontal treatment, except TNF-α, which decreased after performing the treatment. The differences between the treatment group and the control group were not significant for any of the three visits. Most authors agree that treatment of chronic periodontitis does not significantly change levels of serum markers of acute-phase inflammatory and vascular response [41]. It has been suggested that non-surgical mechanical periodontal treatment during pregnancy successfully reduces periodontal inflammation and gingival crevicular fluid cytokine levels, but it has no significant impact on serum biomarkers [38,42]. The complex immunological events that occur during pregnancy may limit the ability to evaluate the systemic effect of periodontal therapy, because studies have shown that pregnant women have a higher production of cytokines depending on the gestational period and the outcome of pregnancy [42].

Regarding the presence of periodontopathogens, the non-surgical periodontal treatment performed in the second trimester reduced the number of all the subgingival periodontal pathogens analyzed and the total bacterial load. These findings indicate that the periodontal therapy of pregnant women with periodontitis produces quantitative and qualitative changes in the microbiota similar to those observed in non-pregnant women [43]. Pregnant women with periodontitis harbor a very complex and pathogenic subgingival microbiota, including a high prevalence of *Fusobacterium nucleatum, Prevotella intermedia, Porphyromonas gingivalis,* and *Parvimona micra* [9]. Martínez-Martínez et al. [44] did not find association between periodontal bacteria and premature birth. In opposition to this, Santa Cruz et al. [9] found a significant association between the presence of *Eikenella corrodens* with preterm birth and the presence of *Capnocytophaga* spp. with low birth weight. Novak et al. [43] showed that basic periodontal therapy significantly reduced the levels of periodontal pathogens but basal levels of these were not associated with premature birth.

The present study is not exempt from a number of methodological limitations that should be considered when extrapolating our results. Up to date, available evidence does not provide clinical, microbiological, or immunological parameters that define patients whose pregnancy outcomes would be improved with periodontal treatment [10]. The selected study group of our study consisted of Caucasian women between 18 and 40 years old and according to the literature the most affected population by periodontitis is the African-American population. The periodontitis definition that we have applied in contrast to previous papers is the one recently published by the EFP-AAP Consensus Meeting [34]. Based on epidemiological and plausibility studies, various treatment strategies could be evaluated that consider specific target populations, as well as timing and intensity of treatment [10].

## 5. Conclusions

We can conclude that non-surgical periodontal treatment in Caucasian patients with periodontitis stage II grade B did not significantly reduce the risk of adverse pregnancy outcomes.

## Figures and Tables

**Table 1:** Demographic description of the study population.

-	Test Group (*n* = 20)	Control Group (*n* = 20)
Age (years, mean ± SD)	32.14 ± 4.27	32.25 ± 4.21
Body Mass Index (Kg/m^2^, mean ± SD)	24.89 ± 5.68	24.73 ± 3.71
Education level (*n*, %)		
Primary	30%	35%
Secondary	40%	35%
University	30%	30%
Residence (*n*, %)		
Rural	75%	90%
Urban	25%	10%
Marital status (*n*, %)		
Single	0%	5%
Common-law partner	40%	40%
Married	60%	55%
Previous spontaneous abortion (*n*, %)		
No spontaneous abortion	95%	95%
One spontaneous abortion	5%	5%
Previous pregnancy (*n*, %)		
No previous pregnancy	80%	85%
One previous pregnancy	20%	15%

**Table 2:** Evolution of clinical parameters during pregnancy.

Clinical Parameters		1st Trimester	2nd Trimester	3rd Trimester
Number of teeth (mean ± SD)	Test group (*n* = 20)	26.95 ± 1.21	26.95 ± 1.21	26.95 ± 1.21
	Control group (*n* = 20)	26.32 ± 2.77	26.32 ± 2.77	26.32 ± 2.77
		*p* = 0.32; CI (−1.91–0.64)	*p* = 0.32; CI (−1.91–0.64)	*p* = 0.32; CI (−1.91–0.64)
Plaque index (mean ± SD)	Test group (*n* = 20)	41.39 ± 18.27	40.21 ± 18.54	21.17 ± 15.59
	Control group (*n* = 20)	32.96 ± 20.08	34.36 ± 18.68	35.68 ± 17.64
		*p* = 0.54; CI (−15.69–8.34)	*p* = 0.32; CI (−17.61–5.91)	*p* = 0.01; CI (3.99–25.01)
Bleeding on probing (mean ± SD)	Test group (*n* = 20)	48.30 ± 14.80	54.57 ± 18.61	27.88 ± 15.29
	Control group (*n* = 20)	32.78 ± 14.92	42.59 ± 18.00	43.21 ± 20.77
		*p* = 0.01; CI (−23.81–−7.89)	*p* = 0.04; CI (−23.55–−0.40)	*p* = 0.01; CI (3.83–26.81)
Probing depth ≤3 mm (%, mean ± SD)	Test group (*n* = 20)	57.38 ± 21.28	52.42 ± 21.43	70.34 ± 17.46
	Control group (*n* = 20)	74.15 ± 12.20	70.00 ± 14.54	65.22 ± 16.81
		*p* = 0.01; CI (3.03–22.92)	*p* = 0.01; CI (5.95–29.21)	*p* = 0.35; CI (−15.95–5.72)
Probing depth 4–5 mm (%, mean ± SD)	Test group (*n* = 20)	35.64 ± 17.34	39.88 ± 19.12	24.39 ± 12.30
	Control group (*n* = 20)	24.69 ± 10.36	26.11 ± 10.67	29.87 ± 12.96
		*p* = 0.09; CI (−15.77–1.10)	*p* = 0.01; CI (−23.62–−3.92)	*p* = 0.17; CI (−2.50–13.46)
Probing depth ≥6 (%, mean ± SD)	Test group (*n* = 20)	6.90 ± 9.59	7.69 ± 10.55	5.31 ± 8.87
	Control group (*n* = 20)	3.68 ± 1.17	4.27 ± 6.95	4.77 ± 6.08
		*p* = 0.01; CI (−9.35–−1.72)	*p* = 0.23; CI (−9.10–2.25)	*p* = 0.82; CI (−5.37–4.29)
Clinical attachment loss ≥3 mm (mm, mean ± SD)	Test group (*n* = 20)	0.74 ± 0.38	0.77 ± 0.46	0.69 ± 0.57
	Control group (*n* = 20)	0.42 ± 0.27	0.50 ± 0.32	0.60 ± 0.35
		*p* = 0.01; CI (−0.46–−0.11)	*p* = 0.03; CI (−0.53–−0.18)	*p* = 0.56; CI (−0.39–0.21)

Student’s *t*-test and U Mann–Whitney.

**Table 3:** Evolution of biochemical parameters during pregnancy.

Biochemical Parameters		1st Trimester	2nd Trimester	3rd Trimester
Fibrinogen (mg/dL, mean ± SD)	Test group (*n* = 20)	365.06 ± 55.12	387.06 ± 49.25	447.65 ± 52.27
	Control group (*n* = 20)	352.39 ± 58.17	401.50 ± 75.88	463.61 ± 89.47
		*p* = 0.54; CI (−42.28–22.47)	*p* = 0.28; CI (−18.43–61.76)	*p* = 0.42; CI (−29.59–68.79)
TNF-α (pg/mL, mean ± SD)	Test group (*n* = 20)	7.80 ± 3.87	7.17 ± 3.20	6.97 ± 2.65
	Control group (*n* = 20)	7.00 ± 3.47	7.80 ± 3.61	7.50 ± 2.72
		*p* = 0.99; CI (−2.07–2.06)	*p* = 0.61; CI (−1.52–2.55)	*p* = 0.50; CI (−1.08–2.15)
Interleukin-6 (pg/mL, mean ± SD)	Test group (*n* = 20)	3.04 ± 2.56	2.54 ± 1.33	2.84 ± 1.42
	Control group (*n* = 20)	3.01 ± 2.26	3.05 ± 2.49	2.36 ± 0.97
		*p* = 0.63; CI (−1.55–0.95)	*p* = 0.43; CI (−0.70–1.61)	*p* = 0.33; CI (−1.06–0.37)
Interleukin-8 (pg/mL, mean ± SD)	Test group (*n* = 20)	14.94 ± 19.09	10.24 ± 6.27	11.41 ± 10.26
	Control group (*n* = 20)	8.61 ± 4.55	19.94 ± 20.84	10.89 ± 8.21
		*p* = 0.54; CI (−9.55–5.13)	*p* = 0.27; CI (−4.64–16.25)	*p* = 0.80; CI (−6.15–4.76)

Student’s *t*-test and U Mann–Whitney.

**Table 4:** Proportions of different bacterial species in test and control groups in 2nd and 3rd trimester.

Microbiological Variables	-	2nd Trimester	3rd Trimester	*p*
*Aggregatibacter actinomycetemcomitans* (%, mean ± SD)	Test (*n* = 20)	0.98 ± 3.00	0 ± 0	0.15; CI (−2.33_0.38)
	Control (*n* = 20)	0.30 ± 2.83	0.28 ± 3.12	0.20; CI (−2.51_0.57)
*Porphyromonas gingivalis* (%, mean ± SD)	Test (*n* = 20)	22.97 ± 25.62	3.63 ± 5.14	0.01; CI (−31.17–−7.51)
	Control (*n* = 20)	23.38 ± 18.23	24.59 ± 19.22	0.21; CI (−20.94–5.01)
*Prevotella intermedia* (%, mean ± SD)	Test (*n* = 20)	2.60 ± 1.20	0 ± 0	<0.01; CI (−3.14–−2.06)
	Control (*n* = 20)	2.79 ± 1.42	2.54 ± 1.55	0.59; CI (−2.44–1.45)
*Tannerella forsythia* (%, mean ± SD)	Test (*n* = 20)	3.30 ± 4.67	0 ± 0	<0.01; CI (−5.41–−1.19)
	Control (*n* = 20)	4.69 ± 1.28	4.80 ± 2.34	0.64; CI (−4.56–2.89)
*Parvimona micra* (%, mean ± SD)	Test (*n* = 20)	0.73 ± 1.65	0 ± 0	0.06; CI (−1.48–0.02)
	Control (*n* = 20)	1.68 ± 3.72	1.86 ± 3.02	0.85; CI (−1.11–1.33)
*Campylobacter rectus* (%, mean ± SD)	Test (*n* = 20)	1.72 ± 7.33	0 ± 0	0.30; CI (−5.04–1.60)
	Control (*n* = 20)	1.32 ± 3.72	1.33 ± 3.83	0.32; CI (−5.56–1.93)
*Fusobacterium nucleatum* (%, mean ± SD)	Test (*n* = 20)	1.96 ± 2.68	0 ± 0	<0.01; CI (−3.17–−0.75)
	Control (*n* = 20)	2.18 ± 1.82	2.20 ± 2.30	0.64; CI (−4.56–2.89)
*Capnocytophaga* (%, mean ± SD)	Test (*n* = 20)	0.06 ± 0.26	0 ± 0	0.32; CI (−0.18–0.06)
	Control (*n* = 20)	0.10 ± 0.25	0.12 ± 0.34	0.19; CI (−0.16–0.03)
*Eikenella corrondens* (%, mean ± SD)	Test (*n* = 20)	0.40 ± 1.13	0 ± 0	0.12; CI (−0.91–0.11)
	Control (*n* = 20)	1.31 ± 1.91	1.22 ± 1.23	0.59; CI (−1.39–2.35)
*Eubacterium* spp. (%, mean ± SD)	Test (*n* = 20)	0 ± 0	0 ± 0	-
	Control (*n* = 20)	0 ± 0	0 ± 0	-

Student’s *t*-test and U Mann–Whitney.

**Table 5:** Primary outcomes.

Primary Outcomes		Test Group (*n* = 20)	Control Group (*n* = 20)	Total
Preterm Birth ^†^	No	19	17	36
	Yes	1	3	4
	Total	20	20	40
Low Birth Weight ^‡^	No	19	17	36
	Yes	1	3	4
	Total	20	20	40

2 × 2 Table (†, ‡ OR = 0.28; CI = 0.02–2.98). † Preterm Birth; ‡ Low Birth Weight

**Table 6:** Dependent variable: pregnancy time (weeks). ANCOVA analysis. R Squared = 0.019 (Adjusted R Squared = −0.065).

Variable	Type III Sum of Squares	*p*
Age	0.206	NS
Body Mass Index	0.181	NS
Treatment	1.194	NS

NS: not significant.

**Table 7:** Dependent variable: newborn weight (kg). ANCOVA analysis. R Squared = 0.116 (Adjusted R Squared = −0.040).

Variable	Type III Sum of Squares	*p*
Age	91,359.53	NS
Body Mass Index	1555.01	NS
Treatment	685,694.99	NS

NS: not significant.
